# Supplementary material for: Self-Penetrating Oligonucleotide Derivatives: Features of Self-Assembly and Interactions with Serum and Intracellular Proteins
Source: Pharmaceutics. 2023 Dec 14;15(12):2779. doi: 10.3390/pharmaceutics15122779 (PMC10747088; doi:10.3390/pharmaceutics15122779)
Supplement: Supplementary file 1 [file pharmaceutics-15-02779-s001.zip › pharmaceutics-2752230-supplementary.pdf]

Supplementary Materials

Self-Penetrating Oligonucleotide Derivatives: Features of Self-Assembly and Interactions with Serum and Intracellular Proteins

Irina Bauer, Ekaterina Ilina, Timofey Zharkov, Evgeniya Grigorieva, Olga Chinak, Maxim Kupryushkin, Victor Golyshev, Dmitry Mitin, Alexey Chubarov *, Svetlana Khodyreva and Elena Dmitrienko *

S1. Result of mass-spectrometry of modified oligonucleotides 2
S2. Duplex thermal denaturation experiments 4
S3. Critical aggregation concentration determination by Nile red encapsulation assay 5
S4. Dynamic light scattering measurements 7
S5. Possible secondary structures formed by oligonucleotides under study 7
S6. Kd determination by fluorescence titration experiment 8
S7. Affinity modification of Ku antigen, PARP1 and DNA polymerase β by [32P]5′-dRp-DNAs 8
S8. Electrophoretic analysis of Ku antigen, PARP1, and DNA polymerase β 9
S9. Cross-linking of [32P]-dRp-containing DNAs to PARP1 and HSA 9
S10. Oligonucleotide cytotoxicity studies 10
S11. Penetration of HSA and its complexes with TZD16 and TZD30 oligomers into cells 10

S1. Result of mass-spectrometry of modified oligonucleotides

TZ30 5'- TCC-TGA-CAT-ACT-TGA-TAC-TTA-GAC-ATT-CT*T -3'

Mr [calculated] / Mr [found] = 9546.0 / 9547.3

Ion charge	Calculated	Found
-7	1362.71	1362.9
-8	1192.25	1192.4
-9	1059.67	1059.9
-10	953.60	953.8
-11	866.82	867.0
-12	794.50	794.6
-13	733.31	733.4
-14	680.86	680.9
-15	635.40	635.4

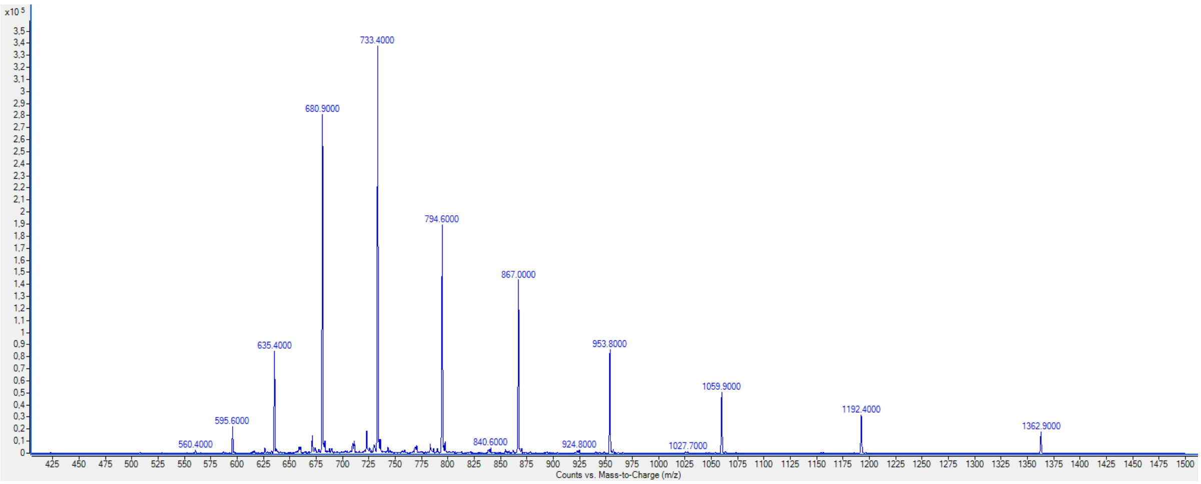

FAM-TZ16 5′-[FAM]CTGACTATGAAGTAT*T-3′

Mr [calculated] / Mr [found] = 5877.5 / 5876.5

Ion charge	Calculated	Found
-4	1468.38	1468.35
-5	1174.50	1174.28
-6	978.58	978.35
-7	838.64	838.35

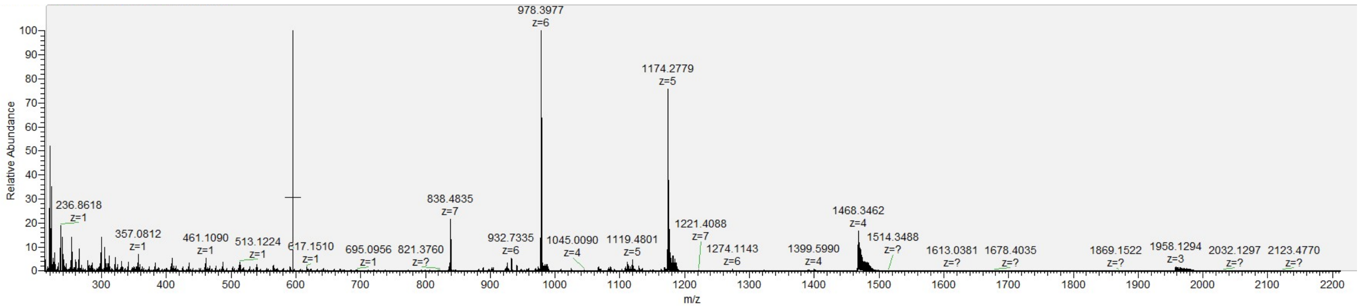

FAM-TZ17 5′-[FAM]AGTCTCGACTTGCTAT*T-3′

Mr [calculated] / Mr [found] = 6130.4 / 6132.0

Ion charge	Calculated	Found
-5	1225.08	1225.3
-6	1020.73	1020.9
-7	874.77	874.9
-8	765.30	765.3
-9	680.16	680.1
-10	612.04	612.0
-11	556.31	556.2

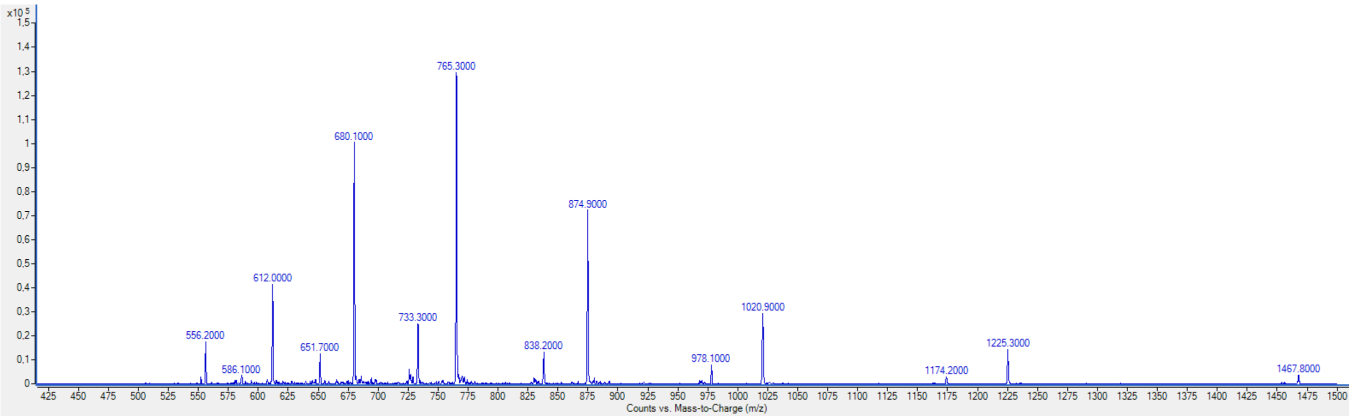

Figure S1. Result of mass-spectrometry of modified oligonucleotides TZ30, FAM-TZ16 and FAM-TZ17.

S2. Duplex thermal denaturation experiments

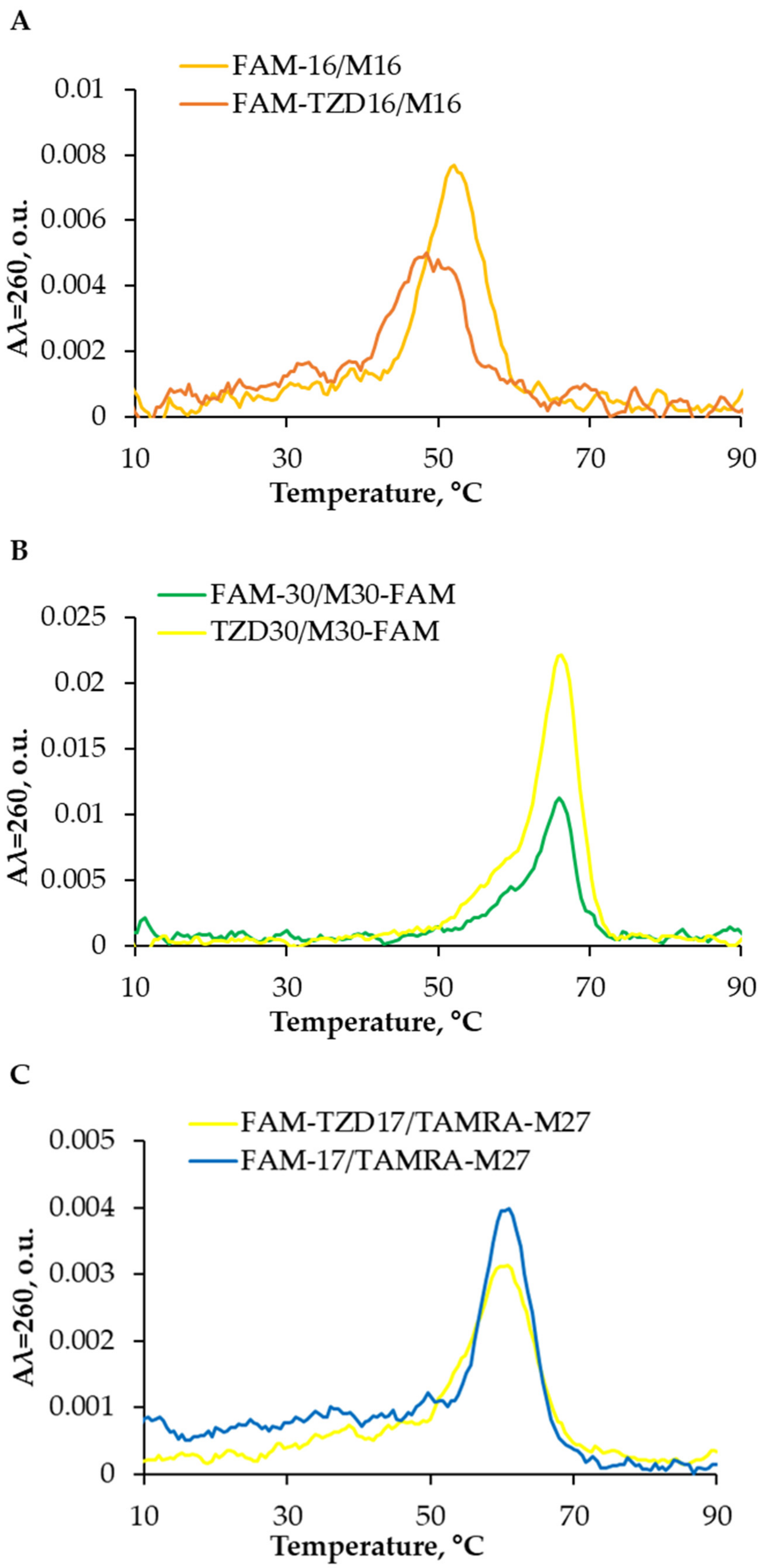

Figure S2. (A–C) Differential melting curves of the duplexes under study; each sample contained 5 µM of a complementary strand in 10 mM NaCac, pH 7.0, and 100 mM NaCl.

S3. Critical aggregation concentration determination by Nile red encapsulation assay

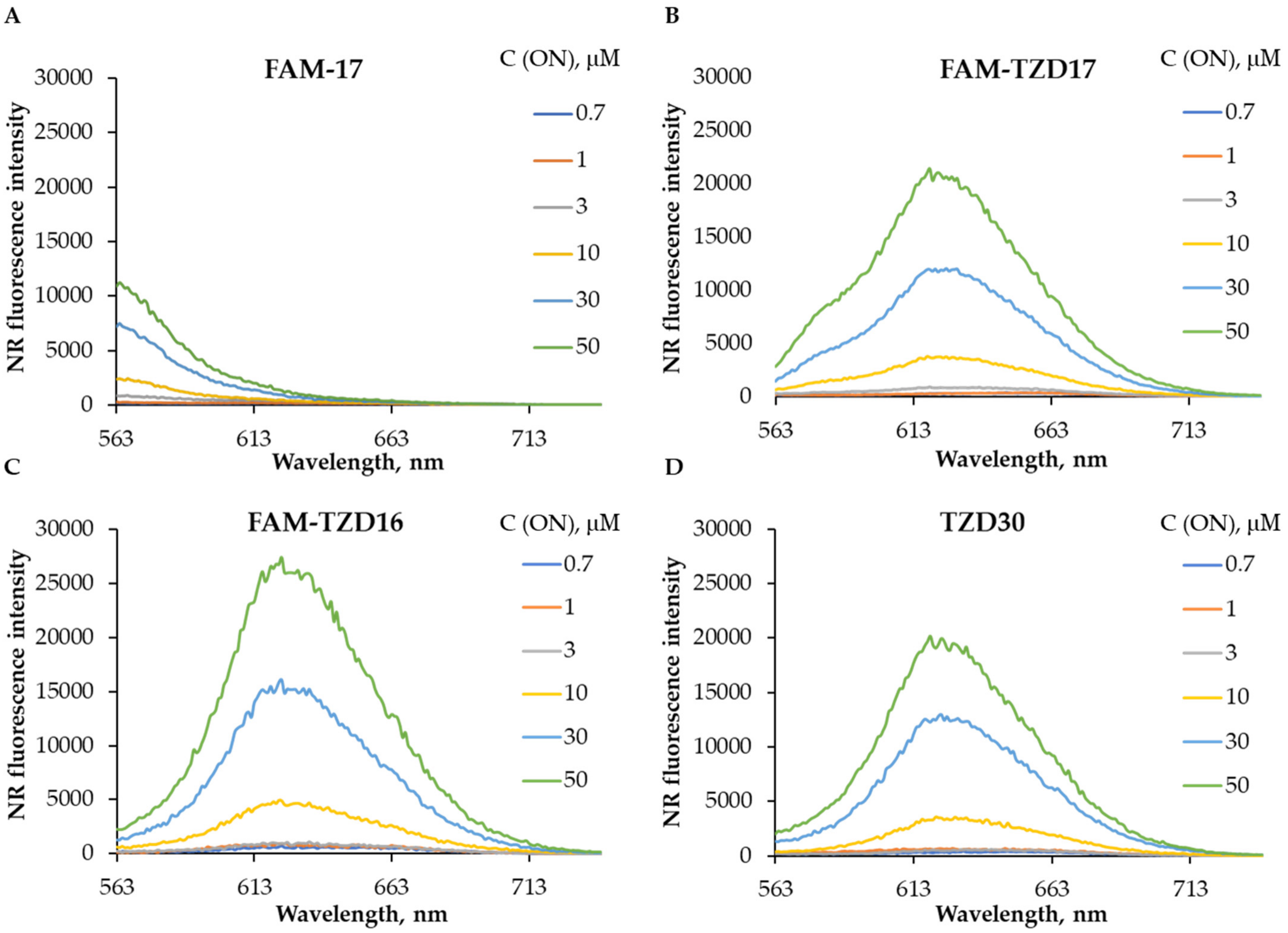

Figure S3. (A–D) Nile Red fluorescence emission spectra in solutions (TAM buffer) of indicated concentrations of oligonucleotides under study.

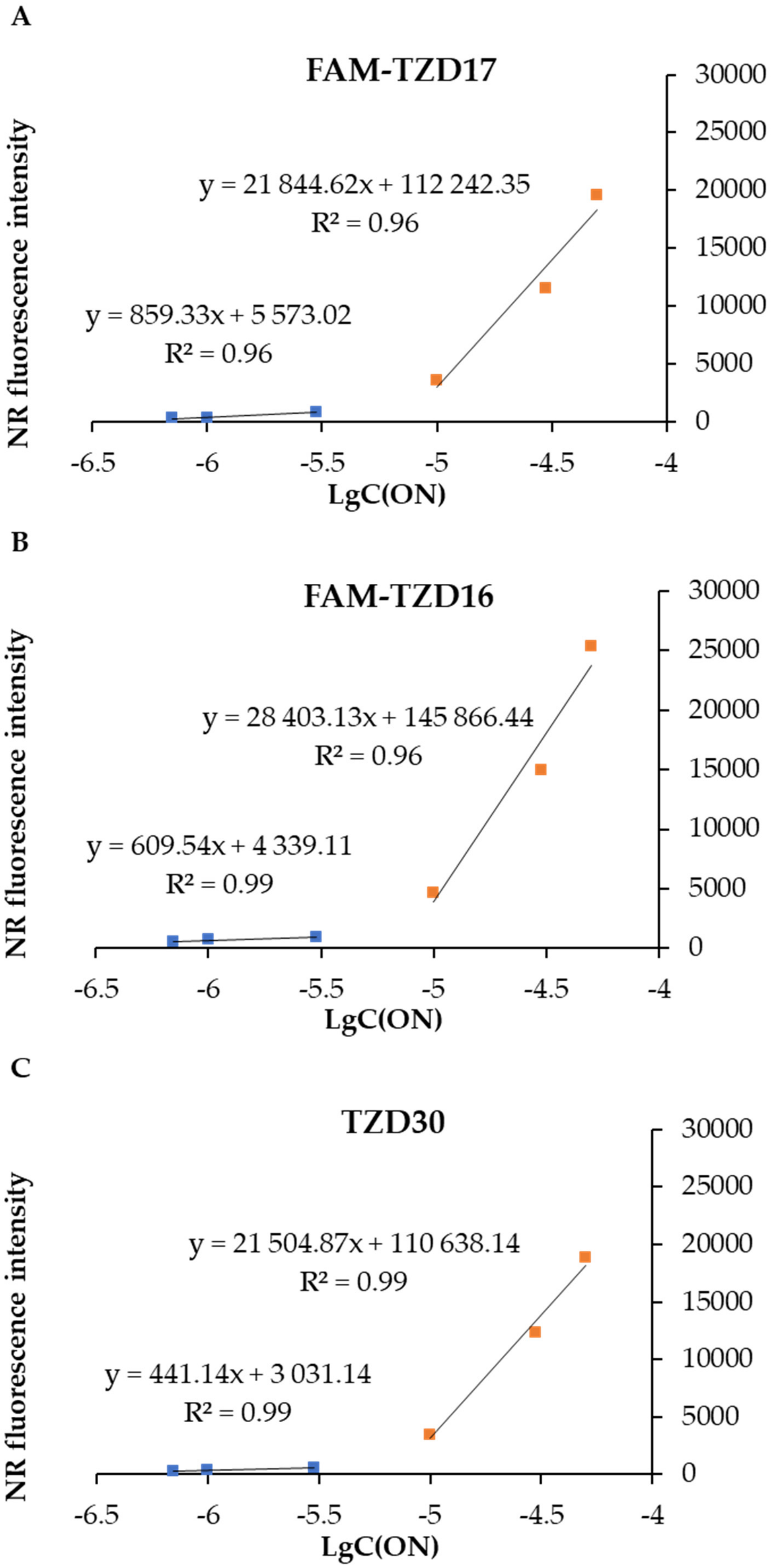

Figure S4. (A–C) Dependence of the Nile Red emission intensity at 630 nm on the logarithm of oligonucleotide concentration (M).

S4. Dynamic light scattering measurements

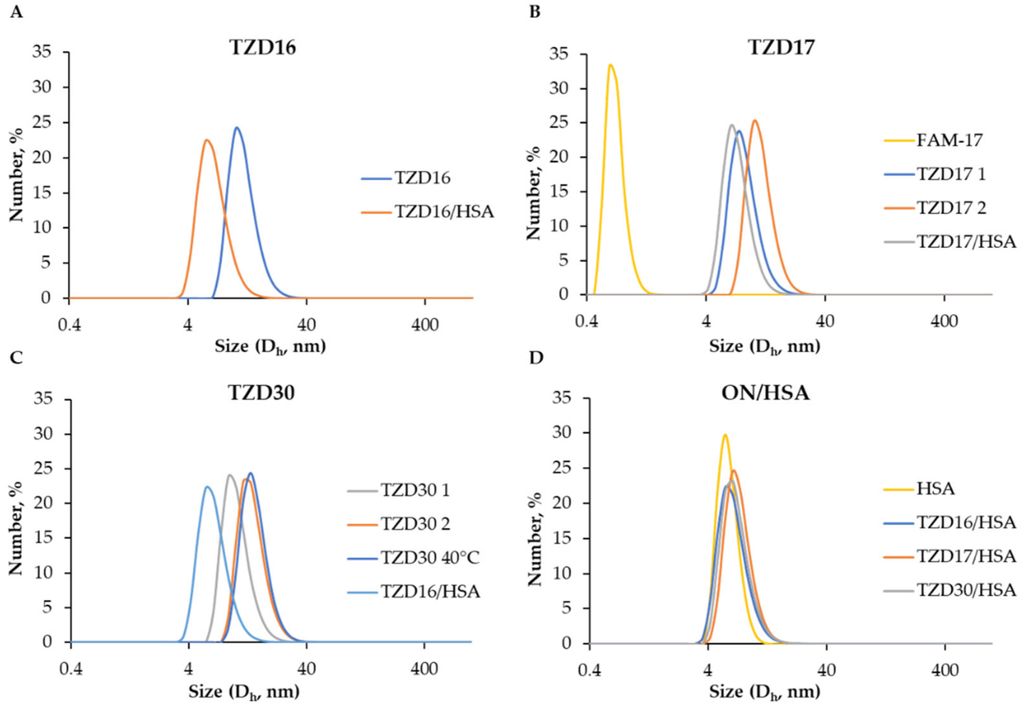

Figure S5. (A–D) Size distribution by number of oligonucleotide particles and HSA–oligonucleotide associates after 12 h incubation in TAM as measured using DLS; oligonucleotide samples contained 10 µM oligomer; ON/HSA ratio in the respective samples is 1:1.

S5. Possible secondary structures formed by oligonucleotides under study

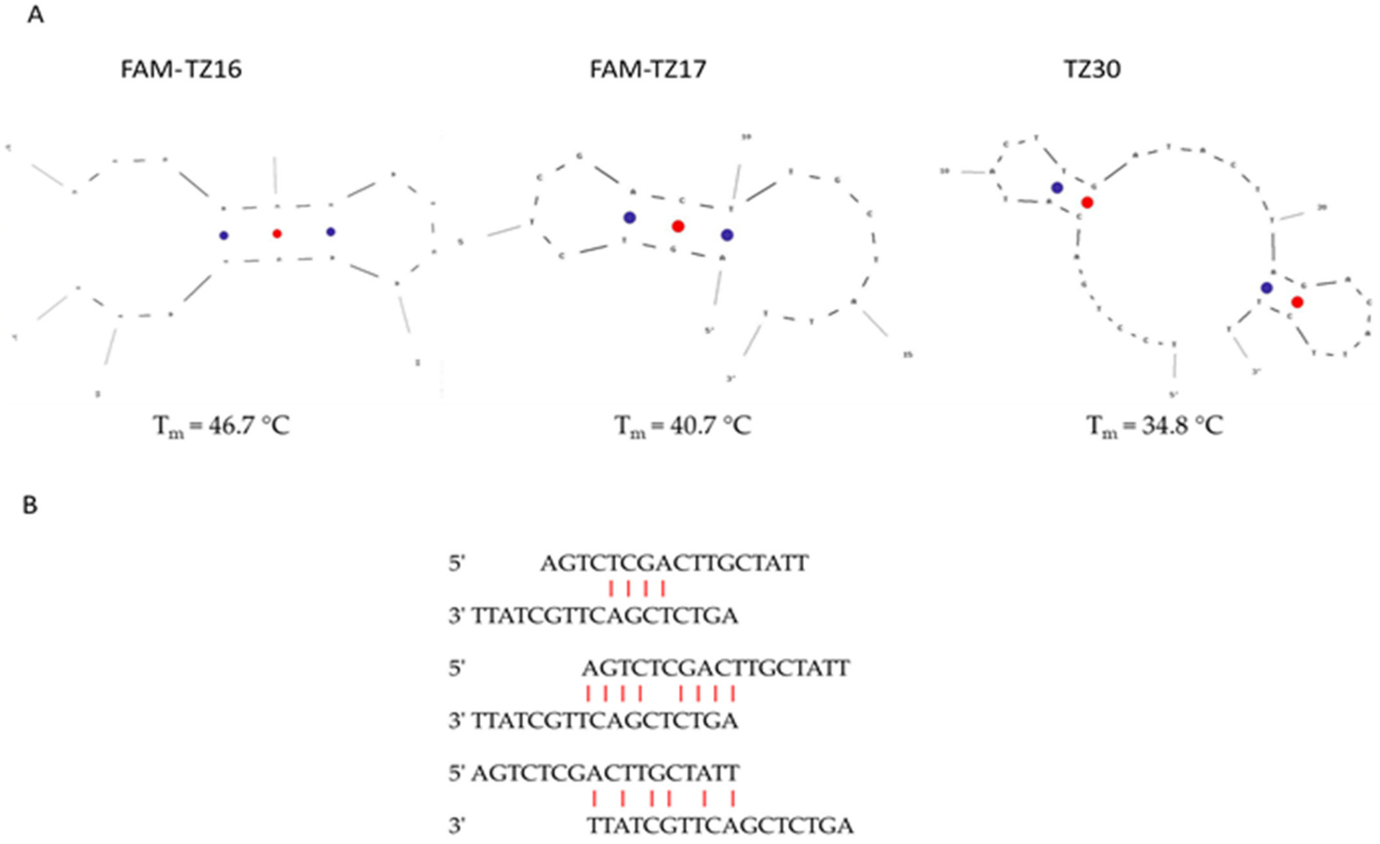

Figure S6. Secondary structures and calculated melting temperatures were predicted using the OligoAnalyzer software lacking the triazinyl phosphoramidate modification (A) and possible structures of FAM-TZ17 self-dimers (B).

S6. Kd determination by fluorescence titration experiment

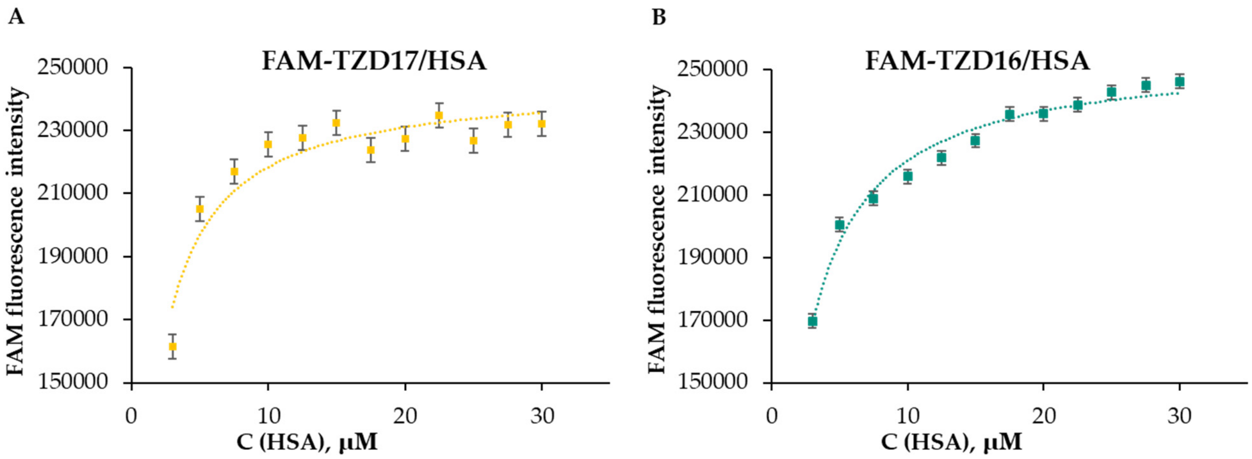

Figure S7. (A,B) Binding curves for the complexes FAM-TZ17/HSA and FAM-TZ16/HSA after 30 min of incubation in PBS buffer, at 37 °C; each sample contained 10 µM oligomer.

S7. Affinity modification of Ku antigen, PARP1 and DNA polymerase β by [32P]5′-dRp-DNAs

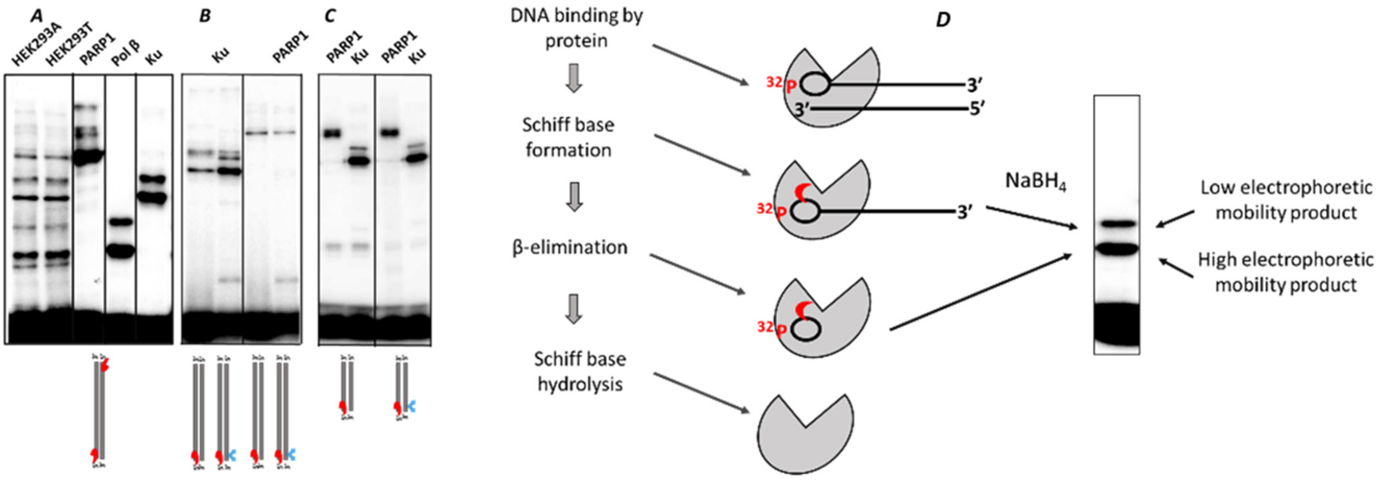

Figure S8. Affinity modification of Ku antigen, PARP1, and DNA polymerase β by [^{32}P]5′-dRp-DNAs. (A)—modification of proteins by the DNA-duplex composed from two 30-mer oligonucleotides bearing a [P^{32}]5′-dRp group at each chain. All reaction mixtures contained 100 nM 5′-dRp-DNA and proteins of whole-cell extracts, 1 mg/ml, or 30 nM Ku antigen, or 150 nM PARP1, or 50 nM DNA polymerase β. The reaction mixtures were incubated at 37 °C for 5 min followed by borohydride treatment for 30 min at 0 °C. The products of DNA–protein cross-linking were separated in 7.5% PAAG according to [97]. (B)—modification of 20 nM Ku antigen, 20 nM PARP1 by 30-mer DNA duplexes. The reaction mixtures were incubated at 37 °C for 10 min followed by borohydride treatment for 30 min at 0 °C. The products of DNA–protein cross-linking were separated in 10% PAAG according to [97]. (C)—modification of 50 nM Ku antigen, 50 nM PARP1 by 16-mer DNA duplexes containing [P^{32}]5′-dRp-oligonucleotide and non-modified or TZD-modified oligonucleotides. The reaction mixtures were incubated at 37 ºC for 10 min followed by borohydride treatment for 30 min at 0 °C. The products of DNA–protein cross-linking were separated in 12.5% PAAG. The gels were dried and exposed to a phosphor imaging screen. Positions of the [^{32}P]5′-dRp group and TZD-modification are shown by red and blue symbols, respectively. (D)—Scheme of [^{32}P]5′-dRp DNA cross-linking to protein and release from covalent complex with protein. The products to be analyzed by SDS-PAAGE were obtained by sodium borohydride treatment of the reaction products.

S8. Electrophoretic analysis of Ku antigen, PARP1, and DNA polymerase β

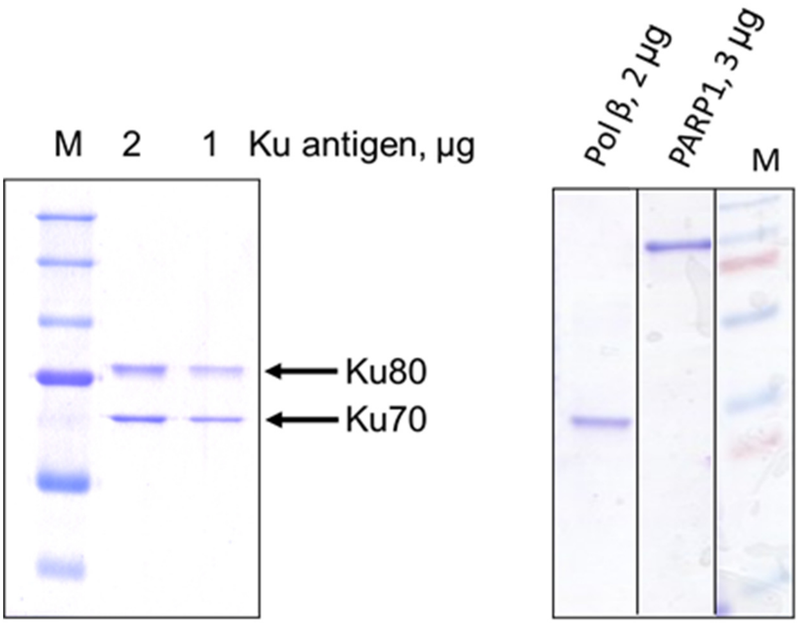

Figure S9. Electrophoretic analysis of used proteins. Purified proteins were analyzed by SDS-PAGE according to the Laemmli method. The gels were 7.5% for Ku antigen and 12.5% for other proteins. The gels were stained with Coomassie R250.

S9. Cross-linking of [32P]-dRp-containing DNAs to PARP1 and HSA

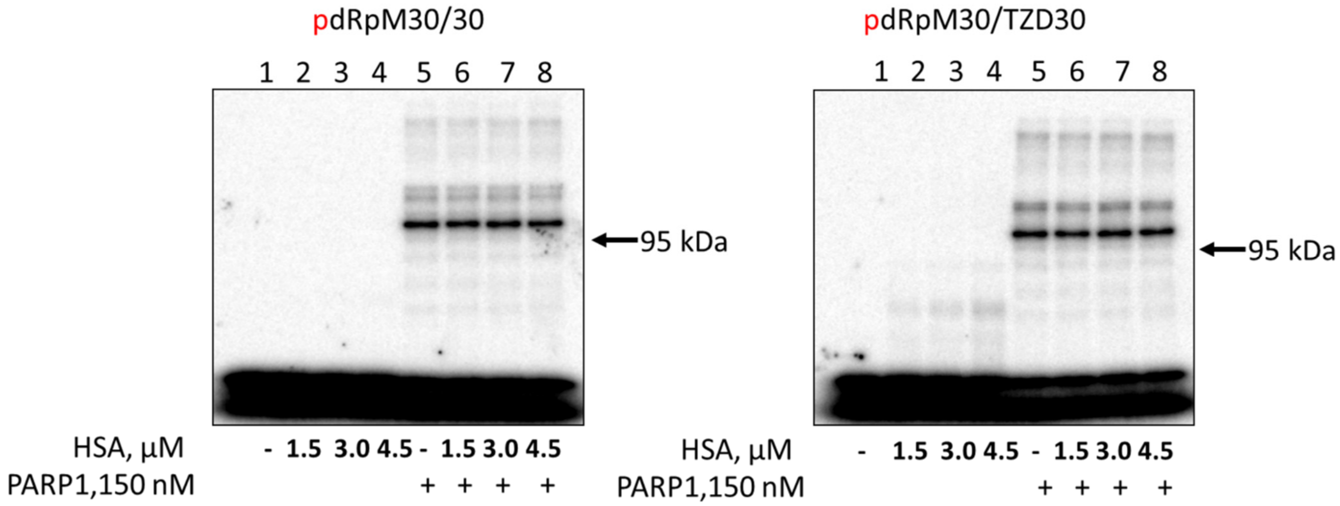

Figure S10. Cross-linking of [^{32}P]-dRp-containing DNAs to PARP1 and HSA. 100 nM [^{32}P]-dRp-containing DNAs were incubated at 37ºC for 10 min with 1.5 μM HSA (lanes 2), 3.0 μM HSA (lanes 3), 4.5 μM HSA (lanes 4), 150 nM PARP1 (lanes 5), 150 nM PARP1+ 1.5 μM HSA (lanes 6), 150 nM PARP1+ 3.0 μM HSA (lanes 7), and 150 nM PARP1+ 4.5 μM HSA (lanes 8). Lanes 1—DNA, control without protein(s). After incubation, the reaction mixtures were supplemented with 20 mM sodium borohydride to reduce the Schiff base for 30 min at 0 °C. The products were resolved in 7.5% SDS-PAAG according to the Laemmli method. The gels were dried and exposed to a phosphor imaging screen. The type of DNA is indicated above the autoradiograph.

S10. Oligonucleotide cytotoxicity studies

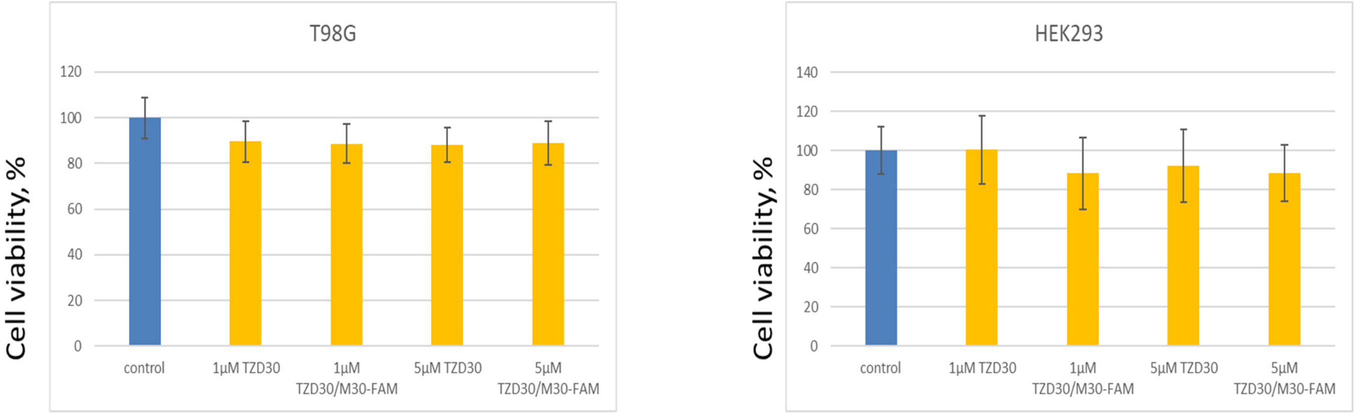

Figure S11. MTT test results for TZD30 and TZD30/M30-FAM on HEK293 and T98G cell lines.

S11. Penetration of HSA and its complexes with TZD16 and TZD30 oligomers into cells

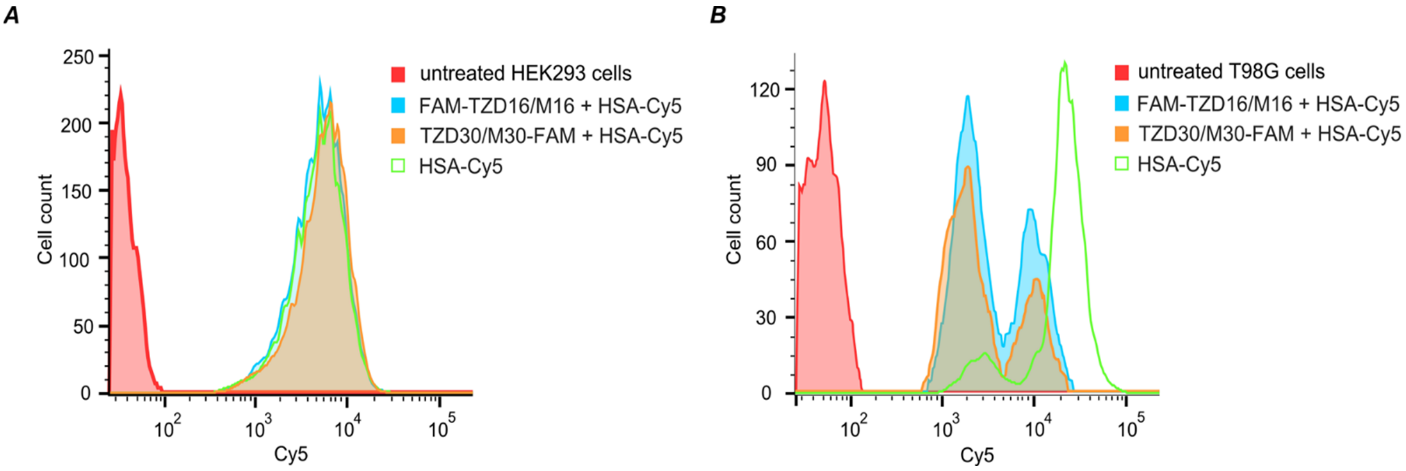

Figure S12. Penetration of HSA and its complexes with TZD16 and TZD30 oligomers into cells. HEK293 (A) and T98G (B) cells were incubated for 4 h with complexes containing 5 µM HSA and 5 µM oligomers.
